# Telemedicine: A New Horizon in Public Health in India

**DOI:** 10.4103/0970-0218.39234

**Published:** 2008-01

**Authors:** Aparajita Dasgupta, Soumya Deb

**Affiliations:** Department of PSM, All India Institute of Hygiene and Public Health, Kolkata, India

## Introduction

Telemedicine is the use of electronic information to communicate technologies to provide and support healthcare when distance separates the participants.(1)

“*Tele*” is a Greek word meaning “distance “and “*mederi*” is a Latin word meaning “to heal”. Time magazine called telemedicine “healing by wire”. Although initially considered “futuristic” and “experimental,” telemedicine is today a reality and has come to stay. Telemedicine has a variety of applications in patient care, education, research, administration and public health.(2) Worldwide, people living in rural and remote areas struggle to access timely, good-quality specialty medical care. Residents of these areas often have substandard access to specialty healthcare, primarily because specialist physicians are more likely to be located in areas of concentrated urban population. Telemedicine has the potential to bridge this distance and facilitate healthcare in these remote areas.(3,4)

## History of Telemedicine

While the explosion of interest in telemedicine over the past four or five years makes it appear as a relatively new use of telecommunications technology, the truth is that telemedicine has been in use in some form or the other for over thirty years. The National Aeronautics and Space Administration (NASA) played an important part in the early development of telemedicine.(5) NASA's efforts in telemedicine began in the early 1960s when humans began flying in space. Physiological parameters were transmitted from both the spacecraft and the space suits during missions.(6)

One of the earliest endeavors in telemedicine, Space Technology Applied to Rural Papago Advanced Health Care (STARPAHC) delivered medical care to the Papago Indian Reservation in Arizona. It ran from 1972–1975 and was conceived by the NASA. Its goals were to provide healthcare to astronauts in space and to provide general medical care to the Papago Reservation.(1) In 1971, 26 sites in Alaska were chosen by the National Library of Medicine's Lister Hill National Center for Biomedical Communication to see if reliable communication would improve village healthcare. It used ATS-1, the first in NASA's series of Applied Technology Satellites launched in 1966. The primary purpose was to investigate the use of satellite video consultation to improve the quality of rural healthcare in Alaska.(7) Since 1977, the Telemedicine Centre at the Memorial University of Newfoundland has worked toward developing interactive audio networks for educational programs and the transmission of medical data.(1) The North-West Telemedicine Project was set up in 1984 in Australia to pilot-test a government satellite communications network (the Q-Network).(1) The project goals were to provide healthcare to people in five remote towns south of the Gulf of Carpentaria. In 1989, NASA conducted the first international telemedicine program, Space Bridge to Armenia/Ufa. Under the auspices of the US/USSR Joint Working Group on Space Biology, telemedicine consultations were conducted using one-way video, voice and facsimile technologies between a medical center in Yerevan, Armenia and four medical centers in the US.(7)

## Definitions and Concepts

### Telemedicine

The World Health Organization (WHO) defines Telemedicine as, “The delivery of healthcare services, where distance is a critical factor, by all healthcare professionals using information and communication technologies for the exchange of valid information for diagnosis, treatment and prevention of disease and injuries, research and evaluation and for the continuing education of healthcare providers, all in the interests of advancing the health of individuals and their communities.”

### Telehealth

Telehealth is the use of electronic information and telecommunications technologies to support long-distance clinical healthcare, patient and professional health-related education and training, public health and health administration.(8)

### Telemedicine Consultation Centre (TCC)

Telemedicine Consulting Centre is the site where the patient is present. In a Telemedicine Consulting Centre, equipment for scanning / converting, transformation and communicating the patient's medical information can be available.(9)

### Telemedicine Specialty Centre (TSC)

Telemedicine Specialty Centre is a site, where the specialist is present. He can interact with the patient present in the remote site and view his reports and monitor his progress.(9)

### Telemedicine System

The Telemedicine system consists of an interface between hardware, software and a communication channel to eventually bridge two geographical locations to exchange information and enable teleconsultancy between two locations.

The hardware consists of a computer, printer, scanner, videoconferencing equipment etc. The software enables the acquisition of patient information (images, reports, films etc.). The communication channel enables the connectivity whereby two locations can connect to each other.(10)

### Utility of Telemedicine [Figure 1](1,9,11–13)

**Figure 1:**
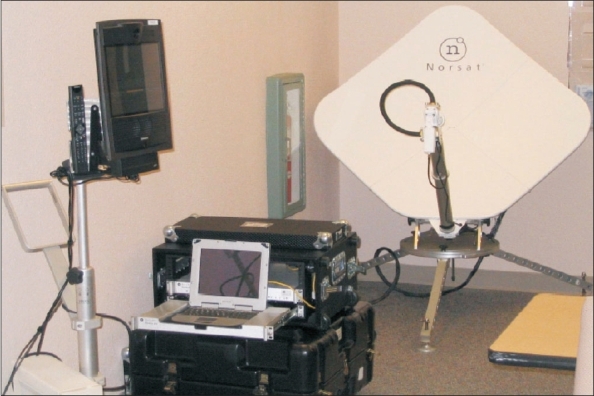
A modern telemedicine system

- Easy access to remote areas
- Using telemedicine in peripheral health set-ups can significantly reduce the time and costs of patient transportation
- Monitoring home care and ambulatory monitoring
- Improves communications between health providers separated by distance
- Critical care monitoring where it is not possible to transfer the patient
- Continuing medical education and clinical research
- A tool for public awareness
- A tool for disaster management
- Second opinion and complex interpretations
- The greatest hope for use of telemedicine technology is that it can bring the expertise to medical practices once telecommunication has been established.
- Telementored procedures-surgery using hand robots
- Disease surveillance and program tracking
- It provides an opportunity for standardization and equity in provision of healthcare, both within individual countries and across regions and continents.
- The Centre for International Rehabilitation recognizes that telecommunication and telemedicine are important technologies to improve and provide rehabilitation services in remote areas. Telemedicine cannot be substitutes for physicians in rural areas especially in developing countries where resources are scarce and public health problems are in plenty. So it is unrealistic to think at this stage of substituting unwilling doctors with this technology. However, it can supplement the current health scenario in a huge way in most countries.

## Types of Technology

Two different kinds of technology make up most of the telemedicine applications in use today. The first, called store and forward, is used to transfer digital images from one location to another. A digital image is taken using a digital camera, ‘stored’ and then sent (‘forwarded’) by a computer to another location. This is typically used for nonemergent situations, when a diagnosis or consultation may be made in the next 24-48 hours and sent back. Teleradiology, telepathology and teledermatology are a few examples.(14)

The other widely used technology, the two-way interactive television (IATV), is used when a ‘face-to-face’ consultation is necessary. The patient and sometimes their provider or more commonly a nurse practitioner or telemedicine coordinator (or any combination of the three), are at the originating site. The specialist is at the referral site, most often at an urban medical center. Videoconferencing equipment at both locations allow a ‘real-time’ consultation to take place.(15) Almost all specialties of medicine have been found to be conducive to this kind of consultation including psychiatry, internal medicine, rehabilitation, cardiology, pediatrics, obstetrics and gynecology and neurology.(15)

## Infrastructure

The telemedicine centers could be broadly classified into the following classes:

Primary Telemedicine Center (PTC)

Secondary Telemedicine Center (STC)

Tertiary Telemedicine Center (TTC)(9)

PTCs would be based in Primary Health Centers, STCs in Secondary Medical Centers and TTCs in Tertiary Medical Centers. The Hardware requirements / standards will be referred in the context of the Telemedicine Consulting and Specialist Centres (TCC) and (TSC).(9)

## Telecommunication Technologies

The first among the challenging questions arising when planning a telemedicine network is ‘What is bandwidth?’ Bandwidth is the capacity that determines how quickly bits may be sent down the channels in a telecommunication medium. Bandwidth is proportional to the complexity of the data for a given level of system performance.(16) The following technologies are currently in use:

## Integrated Services Digital Network (ISDN)

ISDN is a dial-up (not dedicated but used on a call-by-call basis) digital connection to the telecommunication carrier. An ISDN line can carry information at nearly five times the fastest rate achievable using analog modems over POTS (plain old telephone service).(16)

## T-1

This is the backbone of digital service provided to the end user (typically business) in USA today which transmits voice and data digitally at 1.554 megabits per second (Mbps). It can be used to carry analog and digital voice, data and video signals and can even be configured for ISDN service.(16)

## Plain Old Telephone Service (POTS)

POTS transmits data at a rate of up to 56 kilobits per second (kbps) (Bezar 1995) and is the most widely available telecommunication technology in the world. POTS can be suitable for audio conferencing, store-and-forward communication, Internet and low bandwidth videophone conferencing.(16)

## Internet

The Internet has a strong impact in delivering certain kinds of care to patients. In a survey of 1,000 Chief Intelligence Officers (CIOs) conducted by Internet Health Care Magazine, 65% said their organization had a Web presence and another 24% had one in development. With the increasing proliferation of e-health sites on the Web today, many consumers are finding access to online patient scheduling, health education, review of lab work and even e-mail consultations.(16)

## Application of Telemedicine in Public Health

### An epidemiological Surveillance:(17)

Telemedicine applications for epidemiological surveillance are gradually reaching new heights with the development of technology such as geographic information systems (GISs).

- It can give new insight into geographical distribution and gradients in disease prevalence and incidence and valuable insight into population health assessment.
- It also provides valuable information of differential populations at risk based on risk factor profiles.
- It helps in differentiating and delineating the risk factors in the population.
- It also helps in interventional planning, assessment of various interventional strategies and their effectiveness.
- It can play a pivotal role in anticipating epidemics.
- It is an essential tool in real-time monitoring of diseases, locally and globally.
- GIS provides the basic architecture and analytical tools to perform spatial-temporal modeling of climate, environment and disease transmission helpful in understanding the spread of vector-borne diseases. Remote sensing techniques have been recently been used in this regard.
- A GIS-based method for acquiring, retrieving, analyzing and managing data differs from traditional modes of disease surveillance and reporting. It facilitates aggregation and integration of disparate data from diverse sources so it can guide the formulation of public health programs and policy decisions.

### Interactive health communication and disease prevention(17)

Information technology and telemedicine can be used to inform, influence and motivate individuals and population organizations on health, health-related issues and adoption of healthy lifestyles. The various approaches and applications can advance and support primary, secondary and tertiary health promotion and disease prevention agendas.

- It can relay information to individuals as well as to the population as a whole. It can provide an easy access to those living in remote areas.
- It enables informed decision-making. It also simplifies the health decision-making process / or communication between healthcare providers and individuals regarding prevention, diagnosis or management of a health condition. As a result, the users are exposed to a broader choice base.
- It can go a long way to promote and maintain healthy behaviors in the community.
- It can also help in peer information exchange and emotional support. Examples include online Internet applications that enable individuals with specific health conditions, needs or issues to communicate with each other, share information and provide / receive emotional support.
- It promotes self-care and domiciliary care practices. Many living in the remote areas can be benefited by self-management of health problems which will supplement existing health care services.
- It can be a very important tool for the evaluation and monitoring of healthcare services.

## Telemedicine in India

In Utopia, every citizen may have immediate access to the appropriate specialist for medical consultation. In the real world however, this cannot even be a dream. It is a fact of life that “All Men are equal, but some are more equal than others.” We in India are at present, unable to provide even total primary medical care in the rural areas. Secondary and tertiary medical care is not uniformly available even in suburban and urban areas. Incentives to entice specialists to practise even in suburban areas have failed.(18)

In contrast to the bleak scenario in healthcare, computer literacy is developing quickly in India. Healthcare providers are now looking at Telemedicine as their newly found Avatar. Theoretically, it is far easier to set up an excellent telecommunication infrastructure in suburban and rural India than to place hundreds of medical specialists in these places. We have realized that the future of telecommunications lies in satellite-based technology and fiber optic cables.(18)

### The Beginning

The Apollo group of hospitals was a pioneer in starting a pilot project at a secondary level hospital in a village called Aragonda 16 km from Chitoor (population 5000, Aragonda project) in Andhra Pradesh. Starting from simple web cameras and ISDN telephone lines today, the village hospital has a state-of-the-art videoconferencing system and a VSAT (Very Small Aperture Terminal) satellite installed by ISRO (Indian Space Research Organisation). Coupled with this was the Sriharikota Space Center project (130 km from Chennai) which formed an important launch pad of the Indian Space Research Organisation in this field.(2)

## Current Efforts

In India, telemedicine programs are actively supported by:

- Department of Information Technology (DIT)
- Indian Space Research Organization
- NEC Telemedicine program for North-Eastern states
- Apollo Hospitals
- Asia Heart Foundation
- State governments
- Telemedicine technology also supported by some other private organizations(13)

DIT as a facilitator with the long-term objective of effective utilization / incorporation of Information Technology (IT) in all major sectors, has taken the following leads in Telemedicine:

- Development of Technology
- Initiation of pilot schemes-Selected Specialty, e.g., Oncology, Tropical Diseases and General telemedicine system covering all specialties
- Standardization
- Framework for building IT Infrastructure in health(13)

The telemedicine software system has also been developed by the Centre for Development of Advanced Computing, C-DAC which supports Tele-Cardiology, Tele-Radiology and Tele-Pathology etc. It uses ISDN, VSAT, POTS and is used to connect the three premier Medical Institutes of the country (viz. All India Institute of Medical Sciences (AIIMS), New Delhi, Sanjay Gandhi Post Graduate Institute of Medical Sciences (SGPGIMS), Lucknow and Post Graduate Institute of Medical Education and Research (PGIMER), Chandigarh). Now it is being connected to include Medical centres in Rohtak, Shimla and Cuttack.(13)

The telemedicine system has been installed in the School of Tropical Medicine (STM), Kolkata and two District Hospitals. In West Bengal, two hospitals where telemedicine centres have been established are the First Coronary Care Unit inaugurated in Siliguri District Hospital, Siliguri, West Bengal on 24 June, 2001 and Bankura Sammilani Hospital, Bankura, West Bengal inaugurated on 21 July, 2001. Apart from the project at STM, the Second Telemedicine Project has been implemented by Webel ECS at two Referral Centres (Nil Ratan Sircar Medical College and Hospital (NRS MC and H), Kolkata and Burdwan MC and H, Burdwan) and four Nodal Centres (Midnapore (W) District Hospital, Behrampur District Hospital, Suri District Hospital and Purulia District Hospital). The Project uses a 512 kbps leased line and West Bengal State Wide Area Network (WBSWAN) (2 Mbps fiber optic link) as the backbone.(19)

In the past three years, ISRO's telemedicine network has expanded to connect 45 remote and rural hospitals and 15 superspecialty hospitals. The remote / rural nodes include the offshore islands of Andaman and Nicobar and Lakshadweep, the mountainous and hilly regions of Jammu and Kashmir including Kargil and Leh, Medical College hospitals in Orissa and some of the rural / district hospitals in the mainland states.(19)

The Telemedicine project is a “NonProfitable” project sponsored by Rabindranath Tagore International Institute of Cardiac Sciences (RTIICS) Calcutta, Narayana Hrudayalaya (NH) Bangalore, Hewlett Packard, Indian Space Research Organisation (ISRO) and the state governments of the seven North Eastern states of India. The Rabindranath Institute at Kolkata and Narayana Hrudayalaya at Bangalore will be the main Telemedicine linking hub for the seven states. The specialists at both the institutions will offer their services for this project entirely free of charge. A 100 bedded hospital will be identified in each of these seven states and the hospitals will be selected based on distance from the state capital and the lack of a coronary care unit.

In the past two years, the pilot project on Telemedicine in Karnataka has already provided more than 10,000 teleconsultations. In the operational phase, the Karnataka Telemedicine Project is expected to bring multi-specialty healthcare to a significant section of the rural population of Karnataka. This network would serve as a model for the utilization of ‘HEALTHSAT,’ which is proposed for launch in the future.

## Challenges(9,15,19)

- **Perspective of medical practitioners:** Doctors are not fully convinced and familiar with e-medicine.
- **Patients' fear and unfamiliarity:** There is a lack of confidence in patients about the outcome of e-Medicine.
- **Financial unavailability:** The technology and communication costs being too high, sometimes make Telemedicine financially unfeasible.
- **Lack of basic amenities:** In India, nearly 40% of population lives below the poverty level. Basic amenities like transportation, electricity, telecommunication, safe drinking water, primary health services, etc. are missing. No technological advancement can change anything when a person has nothing to change.
- **Literacy rate and diversity in languages:** Only 65.38% of India's population is literate with only 2% being well-versed in English.
- **Technical constraints:** e-medicine supported by various types of software and hardware still needs to mature. For correct diagnosis and pacing of data, we require advanced biological sensors and more bandwidth support.
- **Quality aspect:** “Quality is the essence” and every one wants it but this can sometimes create problems. In case of healthcare, there is no proper governing body to form guidelines in this respect and motivate the organizations to follow-it is solely left to organizations on how they take it.
- **Government Support:** The government has limitations and so do private enterprises. Any technology in its primary stage needs care and support. Only the government has the resources and the power to help it survive and grow. There is no such initiative taken by the government to develop it.

## Conclusion

It does not require too much of a stretch of imagination to realize that telemedicine will soon be just another way to see a health professional. Remote monitoring has the potential to make every minute count by gathering clinical data from many patients simultaneously. However, information may be lost due to a software glitch or hardware meltdown. Therefore, relying too heavily on a computer system to prevent errors in healthcare data may be problematic. There has to be a smart balance between total dependence on computer solutions and the use of human intelligence. Striking that balance may make all the difference in saving someone's life. In 2008, the potential of telemedicine, tele-health and e-health is still left to our imaginations.(20) Time alone will tell that Telemedicine is a “forward step in a backward direction” or to paraphrase Neil Armstrong “one small step for IT but one giant leap for Healthcare”.
